# Supplementary material for: Modeling glioblastoma heterogeneity as a dynamic network of cell states
Source: Mol Syst Biol. 2021 Sep 16;17(9):e10105. doi: 10.15252/msb.202010105 (PMC8444284; doi:10.15252/msb.202010105)
Supplement: Supplementary file 6 — Source Data for Figure 5 [file MSB-17-e10105-s004.zip › Figure5A_sourcedata/GSEA_3071/nosigdb_stateVI.GseaPreranked.1622795169056/IVY_CT.html]

Details for gene set IVY\_CT[GSEA]

|  || Dataset | state63071 |
| Phenotype | NoPhenotypeAvailable |
| Upregulated in class | na\_neg |
| GeneSet | IVY\_CT |
| Enrichment Score (ES) | -0.69149077 |
| Normalized Enrichment Score (NES) | -3.4900239 |
| Nominal p-value | 0.0 |
| FDR q-value | 0.0 |
| FWER p-Value | 0.0 |
Table: GSEA Results Summary

  

Fig 1: Enrichment plot: IVY\_CT      
 Profile of the Running ES Score & Positions of GeneSet Members on the Rank Ordered List

  

| PROBE | GENE SYMBOL | GENE\_TITLE | RANK IN GENE LIST | RANK METRIC SCORE | RUNNING ES | CORE ENRICHMENT || 1 | CDC20 |  |  | 15 | 1.073 | 0.0073 | No |
| 2 | FERMT1 |  |  | 76 | 0.794 | -0.0012 | No |
| 3 | PTTG1 |  |  | 344 | 0.514 | -0.0713 | No |
| 4 | AURKA |  |  | 572 | 0.432 | -0.1309 | No |
| 5 | EGFR |  |  | 669 | 0.407 | -0.1537 | No |
| 6 | CCNB2 |  |  | 967 | 0.350 | -0.2340 | No |
| 7 | S100A3 |  |  | 1588 | 0.270 | -0.4066 | No |
| 8 | LRRC17 |  |  | 1593 | 0.269 | -0.4049 | No |
| 9 | BIRC5 |  |  | 1667 | 0.263 | -0.4227 | No |
| 10 | SDC3 |  |  | 1818 | -0.252 | -0.4625 | No |
| 11 | ID3 |  |  | 1835 | -0.254 | -0.4643 | No |
| 12 | CKAP2L |  |  | 1884 | -0.262 | -0.4750 | No |
| 13 | OLIG2 |  |  | 1968 | -0.274 | -0.4956 | No |
| 14 | MSI1 |  |  | 1982 | -0.275 | -0.4963 | No |
| 15 | GTSE1 |  |  | 2073 | -0.291 | -0.5187 | No |
| 16 | NUF2 |  |  | 2076 | -0.291 | -0.5161 | No |
| 17 | GATM |  |  | 2082 | -0.292 | -0.5144 | No |
| 18 | NPAS3 |  |  | 2184 | -0.312 | -0.5397 | No |
| 19 | CDKN2C |  |  | 2197 | -0.313 | -0.5397 | No |
| 20 | CENPM |  |  | 2287 | -0.330 | -0.5613 | No |
| 21 | BLM |  |  | 2409 | -0.355 | -0.5918 | No |
| 22 | FANCI |  |  | 2411 | -0.356 | -0.5883 | No |
| 23 | PBK |  |  | 2412 | -0.356 | -0.5844 | No |
| 24 | ABAT |  |  | 2438 | -0.365 | -0.5876 | No |
| 25 | H19 |  |  | 2503 | -0.379 | -0.6017 | No |
| 26 | UHRF1 |  |  | 2549 | -0.388 | -0.6102 | No |
| 27 | METRN |  |  | 2617 | -0.404 | -0.6249 | No |
| 28 | SMC4 |  |  | 2634 | -0.408 | -0.6250 | No |
| 29 | KCNIP1 |  |  | 2652 | -0.412 | -0.6254 | No |
| 30 | DSEL |  |  | 2668 | -0.415 | -0.6252 | No |
| 31 | MATN2 |  |  | 2673 | -0.417 | -0.6219 | No |
| 32 | ASF1B |  |  | 2704 | -0.427 | -0.6258 | No |
| 33 | DNMT1 |  |  | 2794 | -0.455 | -0.6461 | No |
| 34 | CENPF |  |  | 2842 | -0.472 | -0.6543 | No |
| 35 | KIF15 |  |  | 2855 | -0.478 | -0.6526 | No |
| 36 | SEMA6A |  |  | 2881 | -0.487 | -0.6545 | No |
| 37 | NCAPG |  |  | 2951 | -0.514 | -0.6685 | No |
| 38 | GAS1 |  |  | 2953 | -0.515 | -0.6632 | No |
| 39 | SOX21 |  |  | 2979 | -0.529 | -0.6647 | No |
| 40 | NES |  |  | 3001 | -0.542 | -0.6648 | No |
| 41 | MEX3A |  |  | 3069 | -0.572 | -0.6776 | No |
| 42 | GINS2 |  |  | 3119 | -0.598 | -0.6851 | Yes |
| 43 | OLIG1 |  |  | 3126 | -0.601 | -0.6803 | Yes |
| 44 | CENPK |  |  | 3134 | -0.610 | -0.6758 | Yes |
| 45 | SCD5 |  |  | 3135 | -0.611 | -0.6692 | Yes |
| 46 | SPATA6 |  |  | 3143 | -0.614 | -0.6646 | Yes |
| 47 | ITGB8 |  |  | 3148 | -0.616 | -0.6592 | Yes |
| 48 | FADS2 |  |  | 3173 | -0.640 | -0.6591 | Yes |
| 49 | MYBL2 |  |  | 3196 | -0.660 | -0.6582 | Yes |
| 50 | NUSAP1 |  |  | 3199 | -0.663 | -0.6517 | Yes |
| 51 | DTL |  |  | 3210 | -0.679 | -0.6473 | Yes |
| 52 | CDCA7 |  |  | 3225 | -0.693 | -0.6438 | Yes |
| 53 | LMNB1 |  |  | 3246 | -0.711 | -0.6418 | Yes |
| 54 | HIST1H2AH |  |  | 3274 | -0.734 | -0.6416 | Yes |
| 55 | SOX9 |  |  | 3280 | -0.739 | -0.6351 | Yes |
| 56 | EZH2 |  |  | 3282 | -0.741 | -0.6274 | Yes |
| 57 | SALL1 |  |  | 3284 | -0.742 | -0.6198 | Yes |
| 58 | DHFR |  |  | 3303 | -0.765 | -0.6167 | Yes |
| 59 | CCNE2 |  |  | 3305 | -0.767 | -0.6087 | Yes |
| 60 | SOX2 |  |  | 3314 | -0.778 | -0.6027 | Yes |
| 61 | RRM2 |  |  | 3317 | -0.783 | -0.5948 | Yes |
| 62 | MLC1 |  |  | 3326 | -0.793 | -0.5886 | Yes |
| 63 | NCAN |  |  | 3329 | -0.794 | -0.5806 | Yes |
| 64 | DBI |  |  | 3349 | -0.812 | -0.5773 | Yes |
| 65 | DDR1 |  |  | 3350 | -0.814 | -0.5686 | Yes |
| 66 | MCM10 |  |  | 3360 | -0.825 | -0.5623 | Yes |
| 67 | NLGN1 |  |  | 3364 | -0.831 | -0.5542 | Yes |
| 68 | RAD51AP1 |  |  | 3375 | -0.848 | -0.5480 | Yes |
| 69 | ETV1 |  |  | 3378 | -0.857 | -0.5393 | Yes |
| 70 | WSCD1 |  |  | 3379 | -0.860 | -0.5301 | Yes |
| 71 | HES6 |  |  | 3384 | -0.864 | -0.5220 | Yes |
| 72 | CDK1 |  |  | 3396 | -0.883 | -0.5156 | Yes |
| 73 | BCHE |  |  | 3405 | -0.909 | -0.5082 | Yes |
| 74 | FBXO5 |  |  | 3413 | -0.919 | -0.5003 | Yes |
| 75 | HMGB2 |  |  | 3414 | -0.922 | -0.4904 | Yes |
| 76 | SOX8 |  |  | 3418 | -0.934 | -0.4812 | Yes |
| 77 | LRRN1 |  |  | 3420 | -0.942 | -0.4714 | Yes |
| 78 | SOX11 |  |  | 3443 | -0.989 | -0.4670 | Yes |
| 79 | ATP6V0E2 |  |  | 3453 | -1.016 | -0.4587 | Yes |
| 80 | TOP2A |  |  | 3457 | -1.020 | -0.4486 | Yes |
| 81 | DLL3 |  |  | 3460 | -1.030 | -0.4381 | Yes |
| 82 | FAM111B |  |  | 3463 | -1.037 | -0.4276 | Yes |
| 83 | KCNQ2 |  |  | 3471 | -1.051 | -0.4183 | Yes |
| 84 | PCDHGC3 |  |  | 3472 | -1.051 | -0.4070 | Yes |
| 85 | ESCO2 |  |  | 3479 | -1.062 | -0.3973 | Yes |
| 86 | TTYH1 |  |  | 3481 | -1.067 | -0.3862 | Yes |
| 87 | TCF12 |  |  | 3494 | -1.100 | -0.3778 | Yes |
| 88 | CLSPN |  |  | 3499 | -1.115 | -0.3669 | Yes |
| 89 | NOVA1 |  |  | 3510 | -1.148 | -0.3575 | Yes |
| 90 | DCLK2 |  |  | 3524 | -1.206 | -0.3482 | Yes |
| 91 | KLHDC8A |  |  | 3552 | -1.370 | -0.3412 | Yes |
| 92 | CKB |  |  | 3579 | -1.575 | -0.3316 | Yes |
| 93 | FAM181B |  |  | 3582 | -1.594 | -0.3151 | Yes |
| 94 | FREM2 |  |  | 3591 | -1.675 | -0.2994 | Yes |
| 95 | HIST1H1B |  |  | 3592 | -1.679 | -0.2814 | Yes |
| 96 | HIST1H1D |  |  | 3596 | -1.757 | -0.2634 | Yes |
| 97 | ASCL1 |  |  | 3604 | -1.822 | -0.2459 | Yes |
| 98 | LRRN3 |  |  | 3606 | -1.871 | -0.2261 | Yes |
| 99 | POU3F2 |  |  | 3610 | -1.933 | -0.2062 | Yes |
| 100 | SCRG1 |  |  | 3613 | -2.165 | -0.1836 | Yes |
| 101 | COL9A3 |  |  | 3618 | -2.307 | -0.1600 | Yes |
| 102 | ID4 |  |  | 3620 | -2.343 | -0.1351 | Yes |
| 103 | C1orf61 |  |  | 3626 | -2.616 | -0.1085 | Yes |
| 104 | PTN |  |  | 3632 | -2.826 | -0.0796 | Yes |
| 105 | IGFBP2 |  |  | 3634 | -3.295 | -0.0446 | Yes |
| 106 | PTPRZ1 |  |  | 3637 | -4.237 | 0.0003 | Yes |
Table: GSEA details [plain text format]

  

Fig 2: IVY\_CT: Random ES distribution      
 Gene set null distribution of ES for **IVY\_CT**
